# Key Characteristics of Residual Malaria Transmission in Two Districts in South-Eastern Tanzania—Implications for Improved Control

**DOI:** 10.1093/infdis/jiaa653

**Published:** 2021-04-27

**Authors:** Fredros Okumu, Marceline Finda

**Affiliations:** 1 Environmental Health and Ecological Sciences, Ifakara Health Institute, Ifakara, Tanzania; 2 School of Public Health, University of the Witwatersrand, Johannesburg, Republic of South Africa; 3 Institute of Biodiversity, Animal Health and Comparative Medicine, University of Glasgow, Glasgow, United Kingdom; 4 School of Life Science and Bioengineering, Nelson Mandela African Institution of Science and Technology, Arusha, Tanzania

**Keywords:** residual malaria transmission, vector control, resistance, *Anopheles funestus*, risk perception, complimentary tools

## Abstract

After 2 decades of using insecticide-treated nets (ITNs) and improved case management, malaria burden in the historically-holoendemic Kilombero valley in Tanzania has significantly declined. We review key characteristics of the residual transmission and recommend options for improvement. Transmission has declined by >10-fold since 2000 but remains heterogeneous over small distances. Following the crash of Anopheles gambiae, which coincided with ITN scale-up around 2005-2012, Anopheles funestus now dominates malaria transmission. While most infections still occur indoors, substantial biting happens outdoors and before bed-time. There is widespread resistance to pyrethroids and carbamates; *An. funestus *being particularly strongly-resistant. In short and medium-term, these challenges could be addressed using high-quality indoor residual spraying with nonpyrethroids, or ITNs incorporating synergists. Supplementary tools, eg, spatial-repellents may expand protection outdoors. However, sustainable control requires resilience-building approaches, particularly improved housing and larval-source management to suppress mosquitoes, stronger health systems guaranteeing case-detection and treatment, greater community-engagement and expanded health education.

Vector control plays a major role in malaria prevention in Africa [1]. Insecticide-treated nets (ITNs) and indoor residual spraying (IRS) in particular have yielded exemplary gains even in areas historically considered holoendemic. Beginning in the 1990s when ITNs were still untreated or hand-treated with insecticides, their impact has increased following the advent of long-lasting insecticide-treated net versions (LLINs) [2]. In many countries, mass distribution campaigns, supplemented by other channels such as social marketing, antenatal clinics, or school-based distribution have enabled near-universal access and equity [3–5].

Before 2000, the south-eastern Tanzanian districts of Ulanga and Kilombero were among the most malarious [6]. Lying in the vast Kilombero river valley between Udzungwa mountains to the north and Mahenge hills to the south, the area experienced intense transmission all year round, with multiple clinical episodes per person per year [7, 8]. Malaria prevalence rates often exceeded 60% in all age groups [7], including new-born children [9], without any observable seasonality in transmission [7]. Interestingly, the observed relationships between parasitemia in humans and transmission intensities suggested that vector control would substantially reduce morbidity [7] without cutting population-level immunity [8]. However, such interventions needed to be extensive and sustained, given the understanding that overall mortality would remain similar between locations with up to 100-fold differences in transmission intensities [10].

Fortunately, these villages benefited from some of the earliest scientific trials of malaria innovations. As a result, access to diagnostics, drugs, and bed nets grew faster than in most other areas, yielding significant gains on mortality, morbidity, and severe malaria cases [11, 12]. Retrospective analysis of surveys conducted in 1990s and 2000s, showed that relative to the high transmission intensities of up to 1400 infectious bites/person/year (ib/p/y) in early 1990s, bed nets achieved 18-fold decrease in transmission, reaching 81 ib/p/y by 2009 [13]. The expanded coverage of ITNs provided an additional 4.6-fold reduction in transmission [13].

Between 2008 and 2010, after LLINs were rolled out in mass campaigns alongside other keep-up campaigns [4, 14], populations of the major malaria vector, *Anopheles gambiae* s.s., started to dwindle [13, 15–17], eventually disappearing from some villages starting in 2012 [13]. This vector species bites mostly indoors and survives mostly on human blood, so ITNs effectively tackled it by reducing access to the preferred vertebrate blood-meal source. At that time, the vectors were also still susceptible, so insecticide treatments dissipated the vector populations faster than they could adapt. By 2015, malaria burden in the region had been cut by approximately 60% relative to the estimated 2000s levels [18]. A recent subnational analysis showed that while parasite prevalence remains high among school-aged children, average malaria prevalence in mothers attending antenatal clinics is approximately 11% in Kilombero district and approximately 19% in Ulanga district [19].

Despite the gains made, the remnant burden is still significant and several challenges have arisen, slowing the progress. Key among these is decline of mosquito susceptibility to public health insecticides [20, 21], possibly due to scale-up of ITNs and agricultural chemicals. Another was drug resistance, which resulted in a change of primary treatment for uncomplicated malaria from chloroquine to sulfadoxine pyrimethamine in 2001, and to artemisinin-based treatments in 2006 [22, 23]. There are also structural and ecological challenges, notably poor housing, which increases the risk of *Anopheles* bites [24], and low household incomes, which confounds health prioritization.

As the malaria control efforts have expanded, Ifakara Health Institute has been monitoring the transmission patterns in Ulanga and Kilombero districts for several years, while also developing and testing tools and approaches to address the gaps. In this review, we summarize the main characteristics of observed malaria transmission events persisting in the area (Figure 1) and outline some options for improved control. The synthesis relies primarily on recent studies in the 2 districts, done between 2008 and 2018 (Table 1), but also draws on data from other sites to explain relevant aspects.

**Table 1.** Studies to Characterize the Residual Malaria Transmission Patterns in Ulanga and Kilombero Districts, South-Eastern Tanzania, and Test Potential New Tools for Improved Control

Category	Activities	Publications
Entomological surveillance	Using different mosquito trapping methods (eg, CDC-Light Traps indoors, SUNA traps outdoors, miniaturized double-net traps, and human landing catches indoors and outdoors, and Prokopack aspirators) to assess densities and characteristics of malaria mosquitoes	[24–31]
	Larval surveys to characterize different aquatic habitats of the dominant malaria vectors	[32]
	Standard WHO insecticide susceptibility tests on field-collected populations of *Anopheles arabiensis* and *An. funestus* to assess presence of and levels of resistance in these populations	[21, 33, 34]
	Experimental hut studies to assess behaviors and responses of the local malaria vector populations to various interventions	[15, 17, 35–37]
	Semifield experiments to assess behaviors of F1 generation of field-collected mosquitoes	Unpublished data
	Laboratory studies to describe key characteristics of field-collected malaria vectors, eg, proportion porous and estimated ages, sibling species identification and *Plasmodium* infection states	[24–29, 35]
Anthropological surveillance	Direct observation of human activities exposing residents to *Anopheles* bites and malaria infections	[29, 38–41]
	Observation of household and environmental characteristics relevant to malaria transmission risk	[24, 25, 29, 41, 42]
	In-depth interviews, focus group discussions, and surveys to assess knowledge, awareness and attitude towards malaria transmission	[29, 38–41, 43]
Testing new malaria control tools	A combination of experimental hut, semifield, and field studies to examine or validate potential of different new complementary tools of malaria surveillance and control	[32, 44–52]

**Figure 1.**
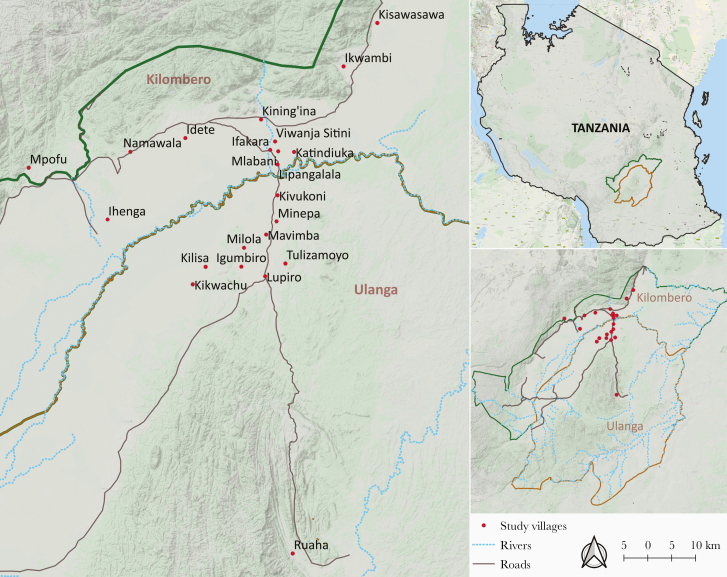
A map of Ulanga and Kilombero districts, south-eastern Tanzania showing villages where the malaria transmission data reviewed here were obtained. Map by Najat Kahamba.

## MALARIA TRANSMISSION IN THE AREA IS NOW PRIMARILY DRIVEN BY *ANOPHELES FUNESTUS* MOSQUITOES

Historical data suggest that *An. gambiae* s.s. dominated malaria transmission in Ulanga and Kilombero districts for many years prior to the mid-2000s, even though *An. funestus* was often the most important vector during the dry season [53]. However, the proportional representation and contribution of *An. gambiae* to malaria transmission, measured as entomological inoculation rates (EIR) began to dwindle after ITNs were introduced [13, 31]. Compared to the early 1990s, biting densities of *An. gambiae* had declined by approximately 20% in early 2000s and by 80% in 2008 [13]. Further declines were observed following large-scale implementation of ITNs, so that by 2012 the species was already undetectable in some villages in Kilombero district [31]. In studies conducted in Ulanga district between 2009 and 2012, polymerase chain reaction (PCR) assays of mosquitoes in the *An. gambiae* complex consistently yielded less than 3% *An. gambiae* s.s., the rest being *An. arabiensis* [15–17, 36]. The *An. gambiae* s.s. populations persisted briefly in villages at the northern tip of Kilombero district till around 2010 [54], before subsequently declining [55].

Many recent studies have confirmed the absence of *An. gambiae* s.s. from most villages across the valley for nearly 10 years, coincident with the period when LLINs have been used at scale [24, 28, 29, 33, 46, 56]. It is highly plausible that the insecticidal bed nets majorly contributed to the decline of this species, which is otherwise highly anthropophilic (prefers biting humans over other vertebrates [57]) and highly endophilic (mostly rests indoors instead of outdoors). However, changes in agricultural practices and ongoing ecosystem modifications may have accelerated this decline. Besides ITN scale-up, the *An. gambiae* declines also coincided with about 60% malaria reduction in the region from 2000 to 2015 [18].

It was thought that another member of the *An. gambiae* complex, that is *An. arabiensis,* would occupy this niche and become the next major malaria vector. However, several studies since then have demonstrated that even in villages where *An. arabiensis* is abundant, its contribution to prevailing transmission is minimal [33]. Instead, *An. funestus*, previously considered important in the dry season because of its unique survival strategies [53], now mediates most malaria infections [33]. Field estimates showed contributions to EIR were already equal between *An. funestus* and *An. arabiensis* from 2011 to 2012 [31]. However, subsequent analysis of data from 2013–2016 illustrated that even in areas where densities of *An. arabiensis* exceeded *An. funestus* by 4:1 ratio, the latter still contributed more than 85% of malaria infections [33]. More recent studies among the migratory farming communities in the valley showed that more than 90% of transmission was mediated by *An. funestus* s.s. Even in the more urban sites such as Ifakara town area and its surroundings, the little transmission persisting there appears to be driven by *An. funestus*. Finda et al observed after 3572 trap nights that the one infected *Anopheles* mosquito was *An. funestus* [29].

There are other *Anopheles* species playing a minor role in transmission. For example, *Anopheles rivulorum*, another member of the wider *An. funestus* group previously described as an important malaria vector [58], has tested positive for *Plasmodium* sporozoites in Kilombero valley [33]. It is however unclear whether these other species can sustain transmission in the absence of *An. funestus*.

These surveys have demonstrated that assessing dominance of malaria vectors in residual transmission settings should be based on *Plasmodium* infection rates rather than relying simply on vector densities and occurrence. For species such as *An. funestus*, which can be cryptic and difficult to find in the aquatic stages yet highly infective even at low densities, detailed surveys should be done before concluding on the importance of different vector species.

The main reasons for *An. funestus* dominance appear to be strong insecticide resistance [34], longer survival as demonstrated by higher proportions of parous females compared to *An. arabiensis* [26], well-adapted dry-season survival strategies [53], and strong preferences for human blood over other animal hosts [57, 59]. The majority of studies in the valley indicate human blood indices above 90% in *An. funestus* [26, 57, 59], although some have showed the mosquitoes can also bite cattle when these hosts are widely available [55, 60, 61].

## MOST MALARIA TRANSMISSION STILL OCCURS INDOORS DESPITE A SIZABLE PROPORTION OF *ANOPHELES* MOSQUITOES BITING OUTDOORS

Outdoor-biting by malaria mosquitoes is a growing concern in the efforts towards malaria elimination [62]. In Africa, infections transmitted outdoors and before bedtime result in more than 10 million excess malaria cases annually [63]. The phenomenon has been known for nearly half a century [64], but methods for estimating its direct impact on malaria control have only recently been optimized [65]. While some mosquitoes naturally bite and rest outdoors [62], the outdoor-biting behavior may also be a response to indoor insecticidal interventions [66–68]. Indeed, after long-term use of ITNs in Ulanga and Kilombero districts, the outdoor-biting proportions of malaria vectors increased, and the overall proportion of *An. arabiensis* mosquitoes [68].

**Figure 2.**
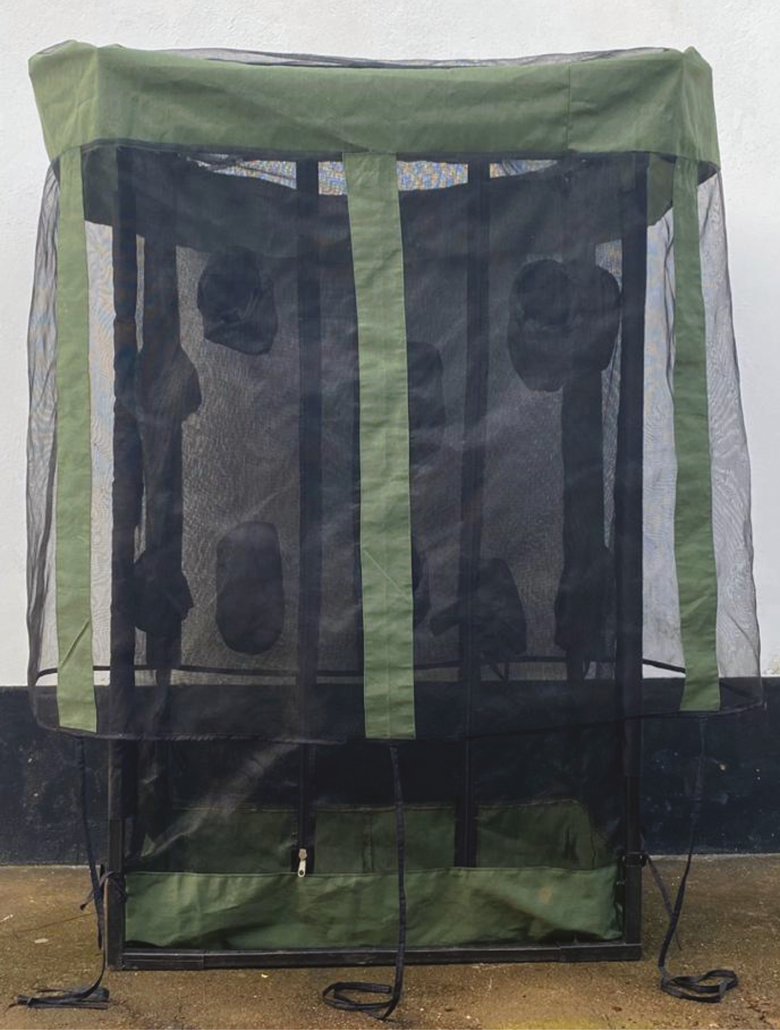
Miniaturized double net trap (DN-Mini) used for sampling host-seeking malaria vectors indoors and outdoors. Detailed description of the DN-Mini is provided by Limwagu et al [46]. The trap allows exposure-free assessment of human biting risk, yielding comparable diversities of mosquito species and physiological states, even though actual densities collected are significantly lower than in standard human-biting collections.

An extended entomological survey was conducted in Ulanga district between 2014 and 2015, where human volunteers sitting indoors or outdoors collected mosquitoes attempting to bite them [28]. This study demonstrated that outdoor-biting proportions were significantly higher than indoors for *An. arabiensis* (68% vs 32%; *P* < .05) [28]. However, there was a smaller difference for *An. funestus* (40% outdoors vs 60% indoors; *P* > .05), indicating that biting by this species is driven strongly by location of humans [28]. Similar observations were made when miniaturized double net traps (DN-Mini) occupied by volunteers (Figure 2) were used indoors and outdoors [46]. More than twice as many *An. arabiensis* were caught outdoors as indoors, but *An. funestus* marginally preferred indoors [46]. A different survey by Finda et al, also using DN-Mini traps, in 8 villages across Ulanga and Kilombero district determined that 71% of *An. arabiensis* mosquitoes were biting outdoors compared to 29% indoors [25]. On the other hand, 64% of host-seeking *An. funestus* were indoors compared to 36% outdoors [25].

**Figure 3.**
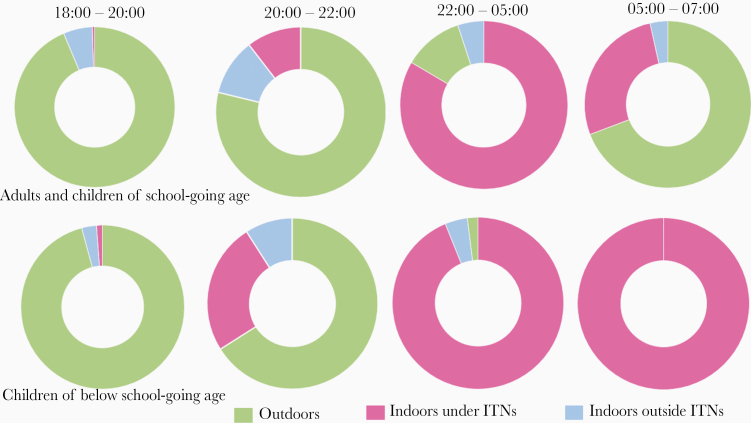
Proportion of household members indoors and outdoors during night hours. A detailed description of this study is provided by Finda et al [25].

The outdoor- or indoor-biting proportions are only relevant if humans are present at the same locations and the same time. Therefore, to assess the actual biting exposures, Finda et al [25] observed human activities indoors and outdoors on an hourly basis (Figure 3). By incorporating the times when individual residents went indoors and the percentage using ITNs, they estimated the overall proportion of exposure occurring indoors to be 63.1% for *An*. *arabiensis* and 78.2% for *An*. *funestus*. Outdoor exposure was high in the evenings between 6 pm and 10 pm (79% of *An. arabiensis* bites and 55% of all *An. funestus* bites), and morning hours from 5 am to 7 am (51% of *An. arabiensis* bites and 71% of *An. funestus* bites) [25]. However, between 10 pm and 5 am, nearly all biting exposure was indoors (96% of all biting by *An. arabiensis* and 99% of all biting by *An. funestus*) [25]. Similar exposure dynamics have been observed in earlier studies by Matowo et al [40] and Moshi et al [38] in the valley. Collectively, these studies show that most exposure to malaria vector bites still happens indoors.

Additionally, studies by Swai et al [30], who examined *Plasmodium* sporozoite infections in the salivary glands of *Anopheles* mosquitoes caught inside and outside migrants’ farm houses, concluded that 78% of all transmission events occurred indoors [30]. Of the indoor transmission events, 91% were mediated by *An. funestus* and the remaining 9% by *An. arabiensis*. All the outdoor infections were by *An. funestus* [30]. An analysis of multiple studies conducted in the 2 districts since 2010 has also showed that if only *Plasmodium*-infected *Anopheles* are considered, nearly all of them were mosquitoes caught indoors, even where up to 70% of the total biting by *Anopheles* mosquitoes was outdoors. In the studies by Limwagu et al [46], mosquitoes collected indoors and outdoors were dissected to compare proportions that were parous and potentially infectious. For *An. funestus*, it was observed that the older potentially infectious subpopulations were mostly indoors and that those biting outdoors were mostly nulliparous [46]. This adds to the evidence that for the vector that carries most malaria in the area, transmission likely happens mostly indoors.

In conclusion, biting exposure and overall likelihood of malaria infection by the main malaria vector *An. funestus* is higher indoors, while for *An. arabiensis*, it is higher outdoors. Given that most of the EIR is from *An. funestus* [26, 29, 30], the prevailing transmission must be occurring mostly indoors, despite the rising risk of outdoor biting.

It has been argued that for malaria elimination to be achieved, this outdoor proportion of biting, though marginal, must also be effectively tackled. However, evidence suggests even mosquitoes that bite outdoors regularly enter houses at least sometime during their adult life [46, 69]. *An. arabiensis* in particular tends to bite outdoors when they are older but indoors when they are younger [46]. When the adult females were dissected by Limwagu et al [46], they observed that although *An. arabiensis* mostly bit outdoors, their older and potentially infectious subpopulations were mostly outdoors. Overall, the available evidence suggests that an effective indoor intervention could tackle both the indoor proportion and outdoor proportion of the main malaria vectors [46, 70].

## LOCAL VECTOR POPULATIONS ARE RESISTANT TO PUBLIC HEALTH INSECTICIDES AND *AN. FUNESTUS* POPULATIONS EXHIBIT THE HIGHEST RESISTANCE INTENSITIES

The primary malaria prevention approach in Tanzania is ITNs, and both Kilombero and Ulanga districts enjoy very high household-level coverage of the intervention [25]. Residents mostly receive nets through government-backed mass distribution every 3–4 years, complemented with keep-up campaigns via antenatal clinics and the school net programs [4, 71]. Due to resource limitation, IRS is currently restricted to northern Tanzania [72–74] and is not done in this area. Unfortunately, as coverage of ITNs has expanded, so has the resistance levels of malaria mosquitoes to the common insecticides [75]. Studies done between 2010 and 2011 already highlighted signs of pesticide resistance in malaria vectors in Kilombero and Ulanga districts [37]. By 2013, both *An. arabiensis* and *An. funestus* from Kilombero district showed low-level resistance to pyrethroids and carbamates, which was increased by 2014 [31]. Studies done around the same time in Ulanga district indicated that *An. arabiensis* was still susceptible to dichlorodiphenyltrichloroethane (DDT), but had signs of pyrethroid resistance, thus requiring closer monitoring [37]. Follow-up studies in multiple villages in mid 2010s demonstrated that resistance in malaria mosquitoes had spread wider, although there were fine-scale variations between villages and seasons [21]. Similar results were observed in tests done on the house mosquito, *Culex pipiens* [20].

Data on *An. funestus* resistance are scarce, partly because of the difficulties of finding wild aquatic populations of these mosquitoes. It therefore rarely appears on national surveys of resistance [75]. Fortunately, some recent studies have used modified protocols, involving wild-caught adults instead of emergent adults from larval collections as recommended by WHO protocols [76]. These studies have demonstrated that both *An. funestus* and *An. arabiensis* now have widespread resistance against DDT, carbamates such as bendiocarb and pyrethroids, including deltamethrin, and permethrin commonly used on ITNs [26, 34]. Kaindoa et al observed that percentage mortalities for *An. funestus* rarely exceeded 30%, suggesting strong resistance in these populations [26]. In 2019, Pinda et al [34], directly compared the resistance levels of *An. funestus* and *An. arabiensis* using WHO protocols for measuring intensity of resistance, and demonstrated that *An. funestus* is indeed more strongly resistant, up to 10 times the standard pyrethroid doses. On the contrary, *An. arabiensis* resistance could be broken at just 5 times the standard doses [34].

The 2011–2012 studies, as well as the more recent ones in 2015–2016, indicated no evidence of any *kdr* mutations usually associated with target-site resistance to DDT and pyrethroids [21, 37]. However, there was a clear reversal of observed resistance levels when mosquitoes were preexposed to the synergist PBO, suggesting a metabolic form of resistance to pyrethroids [21]. Similar reversal using PBO was demonstrated in 2019 on both *An. funestus* and *An. arabiensis* from the 2 districts [34]. The recent studies also demonstrated that nonpyrethroids, such as organophosphates, remain effective against the vectors. Interventions such as ITNs incorporating PBO, or nonpyrethroid IRS (eg, those using organophosphates and neonicotinoids), may thus be effective for vector control here as an immediate response to the resistance challenge.

## TRANSMISSION INTENSITIES HAVE SIGNIFICANTLY DECLINED BUT REMAIN HIGHLY HETEROGENEOUS OVER SMALL DISTANCES

Studies in the 1990s and in early 2000s consistently reported EIR values greater than 300 ib/p/y in villages across Kilombero valley [7, 8, 77]. However, following the widespread scale up of ITNs, ecosystem changes, and subsequent decline of *An. gambiae* s.s populations, transmission intensities have declined significantly. EIR estimates had declined to 78, 51, and 31 ib/p/y by 2008, 2009, and 2011, respectively [31]. More recent studieshave indicated EIRs were already below 20 ib/p/y by 2016; consolidated analyses of Centers for Disease Control and Prevention miniature light trap (CDC-LT) catches and sporozoite infection rates showed unadjusted EIR values of 12 ib/p/y contributed by *An. funestus* and 4 ib/p/y contributed by *An. arabiensis* [26].

Other than the significant declines, the residual malaria transmission intensities are also very evidently heterogeneous over small distances. One study in the migratory farming communities of Kikwachu and Kilisa in Ulanga district (Figure 1) in 2018 and 2019 yielded EIRs as low as 2 ib/p/y [30]. Around the same time, observations from the nearby Tulizamoyo villages estimated EIRs greater than 20 ib/p/y (Ngowo et al unpublished). In a 2015 intensive survey in Ifakara town and surrounding wards (Figure 1), investigators completed 3572 trap-nights but found only one infected mosquito, translating to EIR of just 0.102 ib/p/y [29], nearly undetectable by standard entomological methods.

The observed variations in transmission intensities appear to be correlated with densities of *An. funestus*. Villages at the edge of the valley, which have higher densities of *An. funestus,* have higher transmission than those on the floor of the valley, where the most abundant *Anopheles* is *An. arabiensis* (Ngowo et al unpublished). To verify the geographical heterogeneity in transmission, a parasitological survey was conducted in multiple villages within a 30-km zone, starting from Ifakara town and its surrounding wards in Kilombero district and extending to 12 villages in Ulanga district. Malaria prevalence in all age groups was as low as <1% in Ifakara area but rose steadily to as high as >40% in villages 30 km away (Swai et al unpublished). Subnational estimates from ANC clinics show that malaria parasite prevalence is now 10.7% in Kilombero district and 18.7% in Ulanga district [19], far lower that the pre-ITN levels [8, 9]. In summary, while malaria estimates have been significantly reduced since early 2000s, the residual burden is highly heterogeneous over small distances.

## STRUCTURAL AND ECOLOGICAL RESILIENCE IS IMPORTANT TO SUSTAIN GAINS CATALYZED BY HEALTH COMMODITIES SUCH AS ITNS, DIAGNOSTICS, AND MEDICINES

Alba et al documented wide-ranging impacts of the expanded access to effective malaria prevention and treatment in the Kilombero valley in late 1990s and 2000s [11, 12]. Overall, access to malaria commodities has been high, even for migratory subpopulations that spend weeks or months at distant farms, although home-based treatments for this subgroup may be high and irrational [78].

While the commodities, particularly ITNs have significantly cut malaria burden, structural resilience is required to sustain these gains. This may include improved housing and environmental management to create *Anopheles*-free ecosystems, coupled with expanded access to prompt diagnosis and treatment.

Observational data suggest that malaria transmission risk is higher under poor housing conditions [79, 80]. In south-eastern Tanzania communities, houses with open eaves consistently have more malaria mosquitoes than houses with closed eaves [24, 29, 33, 42]. Analysis of data collected from 2008 to 2011 in Kilombero district concluded that improved housing will be essential to further reduce transmission beyond levels achievable by ITNs [42]. A similar analysis of data from 400 sentinel and randomly selected households in Ulanga district reached a similar conclusion, but added that targeted subsidies could help reduce barriers to safer housing [24]. Unfortunately, most people here still live in houses with open eaves, unscreened windows, or gaps on doors. Although they are fully aware of associated mosquito biting and pathogen transmission risks, their priorities are constrained by low income levels [24]. When Finda et al [29] observed the nearly undetectable EIRs in Ifakara area, they also observed that in addition to high ITN coverage, most houses had brick walls and/or iron roofs (>90%), and 52% had screened windows. On the contrary, in the more rural settings where transmission remains substantial, 51% of houses had mud walls, 75% had open eave spaces, 60% were grass-thatched, 75% had unscreened windows, and 64% left their doors open every evening between 6 pm and 7 pm [24]. The EIR estimates in these villages was 17 ib/p/y [33].

African housing standards are gradually improving, and the proportions of people living in improved homes doubled from 11% in 2000 to 23% in 2015 [81]. Most of this progress appears to be paid for by regular household income and savings, without any government subsidies. If accelerated and incentivized, this trend could build up the necessary structural resilience in communities to protect gains so far accrued from ITNs, drugs, and diagnostics. The commodities are indeed highly impactful, but they require constant replenishments, uninterrupted user compliance, and long-term funding. They are negatively impacted by disruptions in the health systems or supply chains such as is currently seen in the coronavirus disease 2019 (COVID-19) pandemic [82] or during the past Ebola crisis in west Africa [83].

## IT IS CRUCIAL TO INVOLVE THE COMMUNITY MEMBERS IN THE EFFORTS TO SUSTAIN THE GAINS AND EVENTUALLY ACHIEVE MALARIA ELIMINATION

Effective control and elimination of malaria requires significant investment and focus on community knowledge, awareness, and practice. Several qualitative and quantitative studies have indicated relatively high knowledge of malaria transmission among community members in the area [25, 38, 39, 43, 84]. However, the same studies also indicated relatively low perception of risk of malaria transmission, particularly during the early evening and early morning hours; a majority of the community members believe malaria mosquitoes to be mostly active after midnight [25, 38, 39]. This low risk perception is likely because information on the changing dynamics of malaria transmission, that is increasing outdoor and early evening biting, has not been adequately communicated to community members in affected areas. However, the low risk perception could provide a window for human-mosquito contact at times when ITNs are not in use, thereby derailing further efforts towards malaria elimination.

To improve uptake and effectiveness of the current and future malaria control interventions, it is crucial to ensure that the targeted communities have accurate and updated information on the risk of malaria transmission. In a series of focus group discussions with different stakeholders of malaria control in Tanzania, community members in villages in south-eastern Tanzania provided insights into their daily life experiences with respects to malaria transmission and control, and expressed enthusiasm to be involved in the fight against malaria [85]. It is, therefore, also vital to involve the community members in the discussions, development, and implementation of all malaria control strategies.

## THERE ARE CANDIDATE SUPPLEMENTARY INTERVENTIONS THAT COULD POTENTIALLY ACCELERATE ONGOING EFFORTS IN THE SHORT AND MEDIUM TERM

While ITNs have been the main commodity for malaria transmission control, their effectiveness has dwindled in the phase of pyrethroid resistance [34, 37]. They may retain much of their physical protection against mosquito bites but their bioefficacy significantly declines. Recent innovations to create ITNs with PBO [86] or other active ingredients [87, 88] may temporarily reverse these deteriorations, but it is unclear how long the improvements would last as field evidence remains patchy. While trials of PBO nets demonstrated significant improvements in an area with high pyrethroid resistance in northern Tanzania [89], only modest improvements were observed in Burkina Faso [90] and Uganda [91]. Another challenge is the outdoor-biting trend, which presents a small but significant gap beyond the reach of ITNs [25, 65].

While the long-term goal should be to build structural resilience, particularly improved housing, stronger health systems and expanded public education, short- and medium-term efforts may benefit from supplementary tools alongside ITNs or IRS. For best outcomes, the supplementary tools should be those that can control both outdoor-biting and indoor-biting mosquitoes and are also effective against pyrethroid-resistant vector populations. In addition, they should be readily applicable alongside ITNs with minimum user compliance, and affordable pricing.

**Figure 4.**
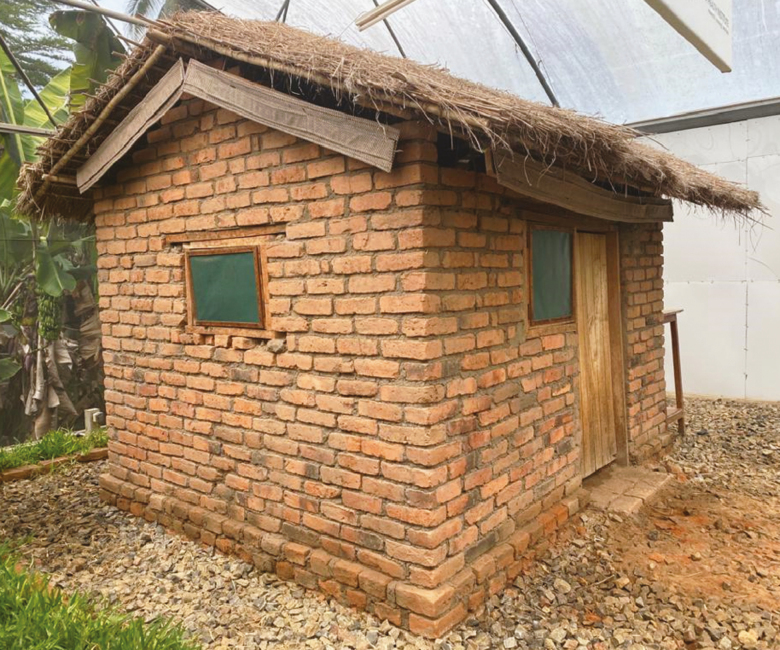
Eave ribbons fitted around the eave space of houses are potentially effective as a complementary intervention against outdoor- and indoor-biting mosquitoes. Detailed description is provided by Mmbando et al [35].

Given the conditions in south-eastern Tanzania and the studies so far completed, some of the options fitting these criteria include eave ribbons treated with spatial repellents such as transfluthrin (Figure 4) [35]. The ribbons protect users both indoors and outdoors, and can be fitted to nearly most houses without completely sealing eave openings. Evidence from experimental hut studies [35] and field studies in migratory households in Ulanga district [30] demonstrated significant efficacies against *An. arabiensis* and *An. funestus*. Other studies have also showed that even nonusers can be protected [50]. Where people spend significant periods of time outdoors in their peridomestic spaces, the ribbons can also be affixed to chairs and outdoor kitchens to complement ITNs [92].

Attractive targeted sugar baits (ATSBs) have also been proposed as a potential option [93, 94]. However, so far, most studies on this technology have been done in dry areas, thus there is inadequate evidence on their potential performance in settings such as Tanzania where the abundant natural vegetation cover could compete with ATSB sugar sources. Future trials should provide greater evidence and also assess whether indoor applications can be effective in Tanzania. Other interventions that may be considered after additional studies include mosquito-repellent shoes for use outdoors and indoors [51], mass drug administration with endectocides such as ivermectin [49, 95], mosquito-assisted larviciding (autodissemination) with pyriproxifen [96], and eave tubes [48].

Another potential approach against pyrethroid-resistant or outdoor-biting mosquitoes is to target them at source. Ecological surveys in Ulanga and Kilombero districts have demonstrated that while the aquatic habitats of the main malaria vector, *An. funestus,* are much rarer than habitats of other mosquitoes, they have well-defined characteristics and are findable, making them a potential target for additional control efforts [32]. In rural south-eastern Tanzania, these habitats are either large permanent or semipermanent ponds with emergent vegetation, small spring-fed pools sometime covered by canopy, or the sides of river streams and tributaries at the edge of the valley [32]. If considered, larval source management for *An. funestus* provides an alternative opportunity for effectively targeting malaria transmission in ways unaffected by prevailing insecticide resistance profiles or outdoor/indoor biting preferences.

Lastly, although IRS is not regularly deployed in south-eastern Tanzania, various nonpyrethroid insecticides remain effective against malaria mosquitoes in the area [21, 34]. As such, high-quality IRS could be deployed where feasible and cost-effective. IRS is a highly effective intervention but is rarely scaled-up because of high costs, logistical difficulties such as the need to remove people’s household belongings before every spraying operation and the need for large trained teams. Innovative ways to simplify and rapidly expand coverage would be crucial. One such option could be to use pretreated materials such as the eave ribbons [35], which would be deployable at scale (Hape et al unpublished), insecticide-treated eave baffles, and window screens, which also require lower quantities of insecticides [52], or insecticide-treated eave-tubes [48]. Further studies should validate the effectiveness of such innovations for complementing ongoing efforts.

## CONCLUSION

As malaria transmission has declined in the south-eastern Tanzanian villages of Ulanga and Kilombero districts, multiple challenges have arisen, compromising efforts towards eventual elimination. Fortunately, our understanding of the transmission ecosystem has vastly improved and we now have new opportunities to accelerate the gains despite the observed challenges. This article has reviewed available evidence from nearly 30 years and described the current status of residual malaria transmission in the area. First, transmission here is now mediated primarily by *Anopheles funestus* mosquitoes even though *An. arabiensis* remains widespread but with very low *Plasmodium*-infection rates. Second, despite a sizable proportion of outdoor-biting by *Anopheles* mosquitoes, most malaria transmission still happens indoors. There is also low perception of risk of outdoor malaria transmission, which could worsen the exposures outside the ITN-protection windows. Third, local vector populations are resistant to pyrethroids and will require alternative insecticide classes to tackle them. We therefore concluded that malaria control efforts should consider the new transmission profile to sustain the gains and accelerate towards elimination. Bed nets remain widespread and protective to users, but their bioefficacy is compromised by the pyrethroid resistance and outdoor biting. In the short and medium term, these gaps could be addressed using high-quality IRS with nonpyrethroids or ITNs incorporating synergists such as PBO. Other complementary interventions may also be added, such as spatial-repellent products targeting outdoor-biting mosquitoes and larval source management against *An. funestus*. However, sustainable control towards elimination will require structural resilience programs, particularly improved housing and environmental management to tackle mosquitoes, stronger health systems guaranteeing effective diagnosis and treatment, greater community engagement, and institutionalized education on malaria control.

## Notes

***Financial support***. This work was supported by the World Health Organization Tropical Disease Research Group (grant number 2015/590235-0); Wellcome Trust Intermediate Fellowship in Public Health and Tropical Medicine (grant number WT102350/Z/13); Howard Hughes Medical Institute-Gates Foundation International Research Scholar Award (grant number OPP1175877 to F. O., Ifakara Health Institute); and Bill and Melinda Gates Foundation (grant number OPP1177156 to Ifakara Health Institute).

***Supplement sponsorship***. The supplement is sponsored by TDR, the Special Programme for Research and Training in Tropical Diseases, based at the World Health Organization.

***Potential conflicts of interest.*** Both authors: No reported conflicts of interest. Both authors have submitted the ICMJE Form for Disclosure of Potential Conflicts of Interest. Conflicts that the editors consider relevant to the content of the manuscript have been disclosed.
